# Household food production is positively associated with dietary diversity and intake of nutrient-dense foods for older preschool children in poorer families: Results from a nationally-representative survey in Nepal

**DOI:** 10.1371/journal.pone.0186765

**Published:** 2017-11-16

**Authors:** Prajula Mulmi, William A. Masters, Shibani Ghosh, Grace Namirembe, Ruchita Rajbhandary, Swetha Manohar, Binod Shrestha, Keith P. West, Patrick Webb

**Affiliations:** 1 Friedman School of Nutrition Science & Policy, Tufts University, Boston, United States of America; 2 Center for Human Nutrition, Department of International Health, Bloomberg School of Public Health, Johns Hopkins University, Baltimore, United States of America; Universidade de Sao Paulo, BRAZIL

## Abstract

**Background:**

Nutrition-sensitive interventions supporting enhanced household food production have potential to improve child dietary quality. However, heterogeneity in market access may cause systematic differences in program effectiveness depending on household wealth and child age. Identifying these effect modifiers can help development agencies specify and target their interventions.

**Objective:**

This study investigates mediating effects of household wealth and child age on links between farm production and child diets, as measured by production and intake of nutrient-dense food groups.

**Methods:**

Two rounds (2013 and 2014) of nationally representative survey data (n = 5,978 observations) were used to measure production and children’s dietary intake, as well as a household wealth index and control variables, including breastfeeding. Novel steps used include measuring production diversity in terms of both species grown and food groups grown, as well as testing for mediating effects of family wealth and age of child.

**Results:**

We find significant associations between child dietary diversity and agricultural diversity in terms of diversity of food groups and of species grown, especially for older children in poorer households, and particularly for fruits and vegetables, dairy and eggs. With each additional food group produced, log-odds of meeting minimum dietary diversity score (≥4) increase by 0.25 (p = 0.01) for children aged 24–59 months. For younger children aged 18–23 months there is a similar effect size but only in the poorest two quintiles of household wealth, and for infants 6–18 months we find no correlation between production and intake in most models.

**Conclusions:**

Child dietary intake is associated with the composition of farm production, most evident among older preschool children and in poorer households. To improve the nutrition of infants, other interventions are needed; and for relatively wealthier households, own farm production may displace market purchases, which could attenuate the impact of household production on child diets.

## Introduction

Children are particularly vulnerable to malnutrition [1,2,3], and stunted growth is especially widespread in poorer and more remote rural households [3,4]. Effects may include negative influences on cognition and health of individuals, and may more broadly adversely affect a national economy [2].

Optimal infant and young child feeding (IYCF) practices include ensuring intake of diverse food groups [5,6], because low diversity is closely linked to inadequate or poor quality of diets [7]. The World Health Organization (WHO) recommends that children under 24 months consume a minimum of 4 out of 7 food groups every day, to raise the probability that a child eats food from both plant and animal sources and promote intake of bioavailable iron, zinc, calcium, and vitamin A among other nutrients [4,5,6,8].

Intake below minimum dietary diversity is widespread in South Asia. In Nepal, for example, the share of children meeting minimum dietary diversity levels increases with age, but from only 12% of infants aged 6–8 months to 22% at 9–11 months, 33% for 12–17 months, and 37% for 18–23 months [9]. This age gradient could be related to differences in child feeding practices, such as older children relying on family meals while younger infants require more frequent feeding with nutrient-dense foods [5,6]. Maternal and child intake of diverse nutrient-dense food groups has been found to be associated with their production on the family’s farm [10,11]. As such, interventions to increase the diversity of farm production have been widely proposed as a path to improved nutrition [12,13,14,15], especially for more remote households who have less ability to use markets to complement their own production with purchased foods [16].

### Motivation

This observational study aims to explore associations between agricultural production diversity and preschool-aged children’s dietary diversity. The rich empirical data used allow for careful consideration of heterogeneity in household wealth and child’s age as potential confounders in such relationships [17]. Both child age and household purchasing power would be expected to modify effects of a household’s own agricultural production on their child’s intake. In economic models of farm households [18,19,20,21], families allocate their available time to caregiving within the household and food production on the farm, using markets to buy and sell whatever products can be exchanged to achieve consumption goals. An important finding from that literature is that food production can potentially be replaced by market purchases, especially in households with sufficient purchasing power and market access to do so [22]. Another significant point is that caregiving within the household, particularly for mothers and infants, requires time that cannot be produced on farm and bought or sold in the market [23]. This suggests that own agricultural production is likely to be associated with child dietary intake only in poorer households without the purchasing power to access markets, with no such association for the youngest children whose dietary intake depends on specialized caregiving efforts in addition to food access.

The hypothesis tests presented here add to the literature on agriculture-nutrition linkages. In Nepal, about two-thirds (68%) of households work in the agricultural sector [24], and isolation, poverty and social conflict may prevent households from using markets to improve dietary intake [1]. This study is also closely linked to recent findings about the high cost of nutrient-dense foods in Nepal [25], and the demonstrated interest of the national government and district officials to achieve the country’s multisector nutrition plan to improve diet quality [26].

**Fig 1:**
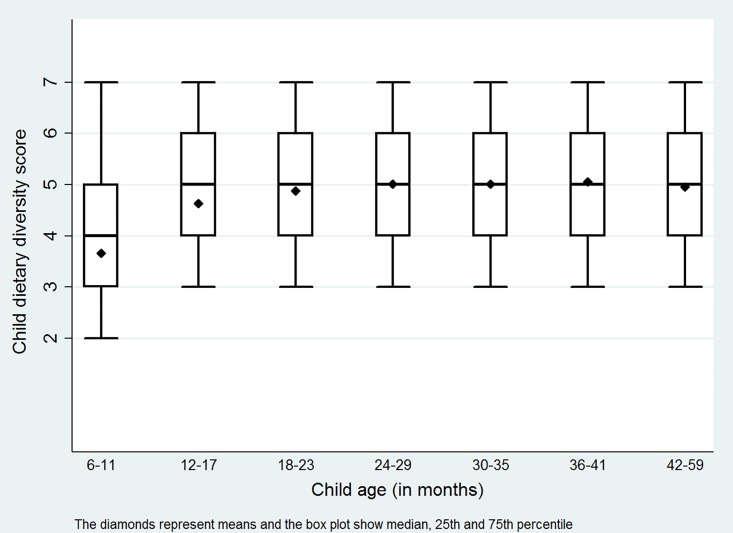
Child dietary diversity by child age (in month). Box plots show median, 25^th^ and 75^th^ percentile of distributions, and diamonds represent mean for each age range.

Our distinctive focus on disaggregation by age, as recommended by the World Health Organization [5], is made possible by the large sample size and repeat visits of the Policy and Science for Health, Agriculture and Nutrition (PoSHAN) surveys conducted in Nepal in 2013 and 2014 [27, 28] as part of a large portfolio of Feed the Future Nutrition Innovation Lab research in that country. As observed in the PoSHAN data, dietary diversity for younger children (6–18 months) is lower than that for older children (≥18 months) (Fig 1). This is consistent with the observation that labor-intensive introduction and frequent feeding of solid foods to younger children is often inadequate [6], while the diets of older children converge to those of adults in the household as soon as they can consume family foods at household meal times.

The aim of this paper is to test the hypothesis that household food production diversity is associated with preschool child dietary intake *only for older children in poorer households*. PoSHAN data were used to determine each household’s agricultural output in terms of the same food groups as those used to measure child diets—this is an important detail that distinguishes this analysis from other studies that have used overall crop/livestock counts as the metric of an agricultural diversity that was unrelated to foods consumed in the same household. We also used counts of individual species of crops and livestock to capture agronomic diversity (to be able to compare with other studies), within as well as a between dietary food groups.

## Methods

### Dataset and study variables

This study uses two waves of panel data obtained from PoSHAN survey conducted in Nepal in 2013 and 2014. Our analysis was reviewed by the Tufts University Institutional Review Board for Social, Behavioral and Educational Research as IRB Study Number 1606018 and excluded from further review on June 14, 2016. Ethical clearance for primary data collection was obtained from the Institutional Review Boards of Johns Hopkins University (USA) and the Nepal Health Research Council (Kathmandu). The survey used multistage stratified cluster sampling to be nationally representative as described in Dorsey et al. (2017) [28]. Interviews occurred during May, June and July of each year, which is typically a period of intermediate food security between harvests [29]. Strata include three agro-ecological zones: Mountains, Hills and Terai, and clusters include seven Village Development Committee (VDCs) per ecological zone [27]. The final sample used in our main results is a balanced panel (n = 2,989 children and 5,978 observations) comprised of children between the ages of 6–59 months and their mothers observed in both years. For this study we exclude caretakers other than mothers, typically grandmothers and fathers, that make up a small subset of the sample (n = 164).

The primary outcome variable of the study is whether children (6–59 months) reach a minimum dietary diversity threshold of four out of seven food groups consumed in the previous week. A secondary set of outcome variables is whether children consume any food from each of seven WHO-defined food groups when that food is produced on the family’s farm. The WHO food groups are (1) grains, roots, and tubers; (2) vitamin-A rich fruits and vegetables; (3) other fruits and vegetables; (4) flesh foods including meat, fish, poultry; (5) eggs; (6) dairy; (7) legumes, nuts and seeds. The WHO’s recommendation is specifically for children under the age of two over the previous 24 hours [5], but that same indicator is used here for all children over 7 days so as to compare dietary trajectories as children age. All dietary data was obtained from a standardized 7-day food-frequency questionnaire, listing over 50 distinct foods that were known from formative research to be commonly or occasionally consumed in Nepal.

Our measures of agricultural production begin with a dichotomous indicator of whether households produced any food at all, and then the total number of food groups grown ranging from 1 to 7. This measure provides a direct counterpart to diversity of dietary intake. To capture agro-ecological diversity within as well as across food groups, we also count the number of distinct crop or livestock species grown. The distribution of that variable is such that many households raise just one or a few of them, while a few have highly diversified farms reporting more than 30 different species of crops and livestock. To transform this measure of agricultural diversity into a variable that might plausibly be associated with child dietary diversity we divide the sample of farmers into quintiles, from 1 to 5. All the variables representing agricultural production decisions are constructed using crops grown during both rainy and dry seasons and livestock raised year-round.

Wealth is measured by an asset index following the same method used by the Demographic and Health Surveys (DHS) [9]. This index enters our hypothesis tests linearly in quintiles of the national distribution. The mediating variable, child age, is used to split the sample to permit full flexibility in all estimated coefficients. Since the optimal IYCF practices only apply to children under two [6], we first divide the sample to compare those aged 6–23 months against those aged 24–59 months. The youngest are further divided into three groups as recommended by the WHO to compare 6–11, 12–17 and 18–23 months [5].

All results control for standard confounding, socio-demographic variables found in the literature. They include caste and ethnicity, religion, maternal education and BMI, mother and child age, whether a child was breastfed, ecological zones, and a total amount of land owned and rented [2,9,30,31,32]. Geographic differences such as distance to markets and disease environments are controlled using VDC fixed effects, and temporal differences such as climatic and market conditions are controlled using year fixed effects. The fixed effects model absorbs all village-specific, year-specific, altitude and climate variation. This approach enables all coefficients to be interpreted as differences among children within villages in any given year, and all analyses are performed using Stata/SE, version 14.

### Estimation strategy

To quantify associations between agricultural production diversity and an indicator of minimum dietary diversity for children (MDDC), logit regression models are estimated using the following base specification:

$${MDDC}_{i}\;=\;\beta_{0}+\beta_{1}{farm}_{ih}+\beta_{2}{wealth}_{ih}+\beta_{3}{farm}_{ih}\times {wealth}_{ih}+\delta Z_{i}+\alpha {VDC}_{i}+\gamma year+\mu_{i\;}$$ (1)

$${MDDC}_{i}\;=\;\beta_{0}+\beta_{1}{fgroup}_{ih}+\beta_{2}{wealth}_{ih}+\beta_{3}{fgroup}_{ih}\times {wealth}_{ih}+\delta Z_{i}+\alpha {VDC}_{i}+\gamma year+\mu_{i\;}$$ (2)

$${MDDC}_{i}\;=\;\beta_{0}+\beta_{1}{fquint}_{ih}+\beta_{2}{wealth}_{ih}+\beta_{3}{fquint}_{ih}\times {wealth}_{ih}+\delta Z_{i}+\alpha {VDC}_{i}+\gamma year+\mu_{i\;}$$ (3)

where MDDC_*i*_ indicates whether a child, *i*, in each of our age groups from 6 to 59 months achieves the minimum dietary diversity score, farm_*ih*_ is a dummy variable equal to 1 when *h* = household of a child *i* produces any food and zero otherwise, fgroup_*ih*_ is a variable ranging from 0–7 indicating number of food groups produced by that household, and fquint_i*h*_ represents quintile of number of crop and livestock species produced (0–5) by that household.

For all the three equations, the study tests the null hypothesis that *β*_1,_ which represents coefficient on the three primary predictor variables, is not statistically significant across the five child age categories. *β*_2_ represents wealth quintile of each child *i* in household *h*. Preliminary analysis indicates that wealth quintile, food group, and agricultural production diversity behave in a linear fashion. These variables are, hence, included in the models in a linear form. *β*_3_ represents an interaction between the predictor variables and wealth, to test hypotheses regarding heterogeneity by the household’s purchasing power.

Z_*i*_ accounts for a vector of control variables at the child, household, and VDC levels as shown in results (Tables 1–9). All the standard determinants of child dietary intake are controlled for in the regressions [33,34]. Block (2006) showed that maternal nutritional knowledge substitutes for maternal schooling in achieving better child nutrition outcomes [35]. Hence, the regression model assumes and accounts for the non-linear relationship between maternal schooling (in years) and child nutrition outcomes. All the logit regressions above include VDC fixed effects and year fixed effects. For each child, *i*, the error term is represented by μ_*i*._ The unbalanced panel data from PoSHAN comprising a larger sample size (Tables 10–11) is used to check the robustness of the results for Eqs 1–3.

**Table 1:** Diet diversity and number of food groups produced by child's age.

	(1) MDDC ≥4 6–11 mo.	(2) MDDC ≥4 12–17 mo.	(3) MDDC ≥4 18–23 mo.	(4) MDDC ≥4 6–23 mo.	(5) MDDC ≥4 24–59 mo.
Food groups grown (0–7)	0.183	-0.086	0.430***	0.139	0.253***
	(0.17)	(0.20)	(0.13)	(0.10)	(0.09)
Wealth quintile (1–5)	0.218	-0.034	0.786***	0.232	0.497***
	(0.31)	(0.34)	(0.20)	(0.18)	(0.19)
Wealth quintile X Food group grown	-0.037	0.088	-0.137***	-0.030	-0.039
	(0.05)	(0.07)	(0.04)	(0.02)	(0.03)
Land rented/used (hectares)	-0.856*	-0.275*	-0.016	-0.075**	-0.014
	(0.51)	(0.17)	(0.03)	(0.03)	(0.02)
Land owned only (hectares)	-0.001	0.176	0.071**	0.047	0.022
	(0.13)	(0.25)	(0.03)	(0.03)	(0.02)
Child (female)	-0.034	0.483	-0.090	0.021	0.128
	(0.24)	(0.47)	(0.27)	(0.18)	(0.10)
Whether breastfed	-1.088	-1.594	-0.772***	-1.354***	-0.033
	(0.84)	(1.33)	(0.29)	(0.18)	(0.06)
Mother’s age (years)	0.031	0.039	0.019	0.034**	0.014*
	(0.02)	(0.03)	(0.02)	(0.01)	(0.01)
Mother’s schooling (years)	0.232*	0.406	0.082	0.168**	-0.005
	(0.12)	(0.27)	(0.16)	(0.08)	(0.07)
Mother’s schooling (squared)	-0.014*	-0.022	-0.001	-0.010**	0.004
	(0.01)	(0.02)	(0.01)	(0.00)	(0.01)
Mother can read	-0.257	-0.800	-0.203	-0.211	0.314
	(0.52)	(0.92)	(0.49)	(0.34)	(0.31)
Mother’s BMI (Kg/m^2^)	-0.048	0.152	-0.015	-0.001	0.049**
	(0.05)	(0.09)	(0.06)	(0.03)	(0.02)
Female head of household	-0.079	-0.246	-0.320	-0.204	-0.389***
	(0.30)	(0.29)	(0.34)	(0.18)	(0.08)
Excluded caste	-0.480**	0.262	-0.896**	-0.515***	-0.737***
	(0.19)	(0.28)	(0.37)	(0.19)	(0.20)
Non-Hindu	0.263	0.250	0.296	0.089	-0.319
	(0.42)	(0.35)	(0.37)	(0.29)	(0.23)
Hill	0.251	-0.224	0.019	0.015	0.843***
	(0.21)	(0.66)	(0.47)	(0.22)	(0.20)
Terai	0.536**	2.024***	1.542***	0.986***	2.463***
	(0.22)	(0.50)	(0.35)	(0.20)	(0.19)
Year (2014)	+	0.161	-0.698***	0.370*	-0.336***
		(0.60)	(0.16)	(0.22)	(0.11)
Constant	0.249	-3.847	-0.357	-0.036	-2.311***
	(0.99)	(2.86)	(1.29)	(0.89)	(0.77)
Observations	396	399	800	1,635	4,343
VDC FE	Yes	Yes	Yes	Yes	Yes

Unit of observation is an individual child between 6–59 months. Coefficients are log-odds ratios. Standard errors in parentheses, clustered on VDCs. All results are from weighted logit regressions with fixed effects for each of 21 VDCs and 2 years. Survey weights are used for children in the balanced panel. The weights are 0.537 for Mountain, 1.711 for Hill and 0.834 for Terai. Description of variables: Food groups (1–7) grown correspond to seven food groups based on WHO and UNICEF’s Minimum Dietary Diversity for Children. Non-farming households (food group = 0) are also included. Whether breastfed is dummy = 1 if children were breastfed in the past seven days and 0 otherwise. Excluded castes are dummy = 1 if Dalit, Janajati and other Terai caste and 0 otherwise. Non-Hindu are dummy = 1 if a household is non-Hindu and 0 if Hindu. Hill and Terai are dummy = 1 and 0 otherwise and dropped if dummy = 1 for Mountain.

^+^ Dropped because of collinearity.

*** p<0.01,

** p<0.05,

* p<0.1

**Table 2:** Diet diversity and quintile of food species produced by child's age.

	(1) MDDC ≥4 6–11 mo.	(2) MDDC ≥4 12–17 mo.	(3) MDDC ≥4 18–23 mo.	(4) MDDC ≥4 6–23 mo.	(5) MDDC ≥4 24–59 mo.
Ag. diversity quintile (0–5)	0.426***	0.257	0.659***	0.344***	0.361***
	(0.16)	(0.40)	(0.23)	(0.11)	(0.11)
Wealth quintile (1–5)	0.274	0.226	0.778***	0.311*	0.426**
	(0.25)	(0.42)	(0.23)	(0.17)	(0.17)
Ag. diversity quintile X Wealth quintile	-0.080	0.018	-0.203***	-0.076***	-0.033
	(0.05)	(0.14)	(0.07)	(0.03)	(0.04)
Land rented/used (hectares)	-0.911*	-0.282*	-0.014	-0.074**	-0.021
	(0.52)	(0.17)	(0.03)	(0.03)	(0.01)
Land owned only (hectares)	-0.044	0.171	0.070**	0.046*	0.016
	(0.12)	(0.26)	(0.03)	(0.03)	(0.02)
Child (female)	-0.021	0.520	-0.077	0.025	0.131
	(0.24)	(0.47)	(0.27)	(0.18)	(0.10)
Whether breastfed	-1.212	-1.585	-0.778***	-1.359***	-0.045
	(0.84)	(1.36)	(0.29)	(0.18)	(0.06)
Mother’s age (years)	0.031	0.032	0.021	0.034**	0.013*
	(0.02)	(0.03)	(0.02)	(0.01)	(0.01)
Mother’s schooling (years)	0.238*	0.413	0.067	0.165**	-0.017
	(0.12)	(0.28)	(0.16)	(0.08)	(0.08)
Mother’s schooling (sq.)	-0.014*	-0.022	0.000	-0.010**	0.005
	(0.01)	(0.02)	(0.01)	(0.00)	(0.01)
Mother can read	-0.342	-0.823	-0.189	-0.233	0.307
	(0.52)	(0.93)	(0.49)	(0.34)	(0.32)
Mother’s BMI (Kg/m^2^)	-0.047	0.155	-0.016	-0.000	0.053***
	(0.05)	(0.10)	(0.06)	(0.03)	(0.02)
Female head of household	-0.018	-0.226	-0.272	-0.156	-0.369***
	(0.32)	(0.31)	(0.36)	(0.19)	(0.09)
Excluded caste	-0.440**	0.456	-0.888**	-0.477***	-0.654***
	(0.18)	(0.35)	(0.36)	(0.17)	(0.18)
Non-Hindu	0.268	0.288	0.347	0.100	-0.279
	(0.42)	(0.38)	(0.36)	(0.28)	(0.23)
Hill	0.423**	-0.213	0.071	0.096	0.923***
	(0.21)	(0.77)	(0.46)	(0.25)	(0.21)
Terai	0.858***	2.348***	1.680***	1.162***	2.663***
	(0.22)	(0.48)	(0.41)	(0.21)	(0.18)
Year (2014)	+	0.172	-0.657***	0.384*	-0.334***
		(0.56)	(0.16)	(0.22)	(0.11)
Constant	-0.279	-4.961*	-0.537	-0.525	-2.486***
	(0.96)	(2.66)	(1.32)	(0.78)	(0.67)
Observations	396	399	800	1,635	4,343
VDC FE	Yes	Yes	Yes	Yes	Yes

Unit of observation is an individual child between 6–59 months. Coefficients are log-odds ratios. Standard errors in parentheses, clustered on VDCs. All results are from weighted logit regressions with fixed effects for each of 21 VDCs and 2 years. Survey weights are used for children in the balanced panel. The weights are 0.537 for Mountain, 1.711 for Hill and 0.834 for Terai. Description of variables: Agricultural production diversity (Ag. div) quintile (1–5) is generated from total count of food species (1–32) produced in a farming household; non-farmers (food quintile = 0) are also included. Food species count is created from a sum of crop species (0–29) and livestock grown (0–6) per household. Range of food species count included in the quintile is as follows: First (1–2), Second (3–5), Third (6–9), Fourth (10–14), and Fifth (15–32). Whether breastfed is dummy = 1 if children were breastfed in the past seven days and 0 otherwise. Excluded castes are dummy = 1 if Dalit, Janajati and other Terai caste and 0 otherwise. Non-Hindu are dummy = 1 if a household is non-Hindu and 0 if Hindu. Hill and Terai are dummy = 1 and 0 otherwise and dropped if dummy = 1 for Mountain.

^+^ Dropped because of collinearity

*** p<0.01,

** p<0.05,

* p<0.1

**Table 3:** Dietary diversity in farm vs. non-farm households by child's age.

	(1) MDDC ≥4 6–11 mo.	(2) MDDC ≥4 12–17 mo.	(3) MDDC ≥4 18–23 mo.	(4) MDDC ≥4 6–23 mo.	(5) MDDC ≥4 24–59 mo.
Farming household	0.090	-1.259	1.288	-0.191	0.757
	(1.22)	(1.51)	(1.37)	(0.64)	(0.58)
Wealth quintile (1–5)	0.065	-0.169	0.752	0.077	0.431**
	(0.43)	(0.58)	(0.57)	(0.24)	(0.20)
Farming household X Wealth quintile	0.014	0.466	-0.620	0.038	-0.125
	(0.35)	(0.53)	(0.57)	(0.16)	(0.16)
Land rented/used (hectares)	-0.785	-0.215	-0.015	-0.076**	-0.012
	(0.51)	(0.15)	(0.04)	(0.03)	(0.02)
Land owned only (hectares)	0.024	0.342	0.066**	0.047*	0.025
	(0.12)	(0.27)	(0.03)	(0.03)	(0.02)
Child (female)	-0.054	0.511	-0.066	0.018	0.144
	(0.23)	(0.47)	(0.26)	(0.17)	(0.10)
Whether breastfed	-1.036	-1.636	-0.790***	-1.356***	-0.047
	(0.86)	(1.34)	(0.27)	(0.19)	(0.07)
Mother’s age (years)	0.034	0.043	0.022	0.036**	0.017**
	(0.02)	(0.03)	(0.02)	(0.02)	(0.01)
Mother’s schooling (years)	0.248**	0.402	0.106	0.171**	-0.002
	(0.12)	(0.27)	(0.17)	(0.08)	(0.07)
Mother’s schooling (squared)	-0.015*	-0.022	-0.003	-0.010**	0.004
	(0.01)	(0.02)	(0.01)	(0.00)	(0.01)
Mother can read	-0.256	-0.783	-0.161	-0.186	0.327
	(0.55)	(0.92)	(0.49)	(0.34)	(0.31)
Mother’s BMI (Kg/m^2^)	-0.051	0.148	-0.014	-0.001	0.049**
	(0.05)	(0.10)	(0.06)	(0.03)	(0.02)
Female head of household	-0.096	-0.238	-0.403	-0.244	-0.445***
	(0.30)	(0.31)	(0.36)	(0.19)	(0.08)
Excluded caste	-0.440***	0.297	-0.915**	-0.523***	-0.739***
	(0.17)	(0.26)	(0.37)	(0.19)	(0.20)
Non-Hindu	0.269	0.150	0.343	0.076	-0.354
	(0.44)	(0.34)	(0.38)	(0.30)	(0.25)
Hill	0.144	-0.141	-0.278	-0.034	0.891***
	(0.22)	(0.65)	(0.45)	(0.21)	(0.20)
Terai	0.285	1.710***	1.257***	0.810***	2.108***
	(0.20)	(0.47)	(0.32)	(0.14)	(0.14)
Year (2014)	+	0.144	-0.673***	0.383*	-0.314***
		(0.57)	(0.15)	(0.21)	(0.11)
Constant	0.917	-2.708	0.433	0.820	-1.749**
	(1.21)	(3.63)	(1.25)	(0.91)	(0.87)
Observations	396	399	800	1,635	4,343
VDC FE	Yes	Yes	Yes	Yes	Yes

Unit of observation is an individual child between 6–59 months. Coefficients are log-odds ratios. Standard errors in parentheses, clustered on VDCs. All results are from weighted logit regressions with fixed effects for each of 21 VDCs and 2 years. Survey weights are used for children in the balanced panel. The weights are 0.537 for Mountain, 1.711 for Hill and 0.834 for Terai. Description of variables: Farm households are dummy = 1 if households produce any food and 0 otherwise. Whether breastfed is dummy = 1 if children were breastfed in the past seven days and 0 otherwise. Excluded castes are dummy = 1 if Dalit, Janajati and other Terai caste and 0 otherwise. Non-Hindu are dummy = 1 if a household is non-Hindu and 0 if Hindu. Hill and Terai are dummy = 1 and 0 otherwise and dropped if dummy = 1 for Mountain.

^+^ Dropped because of collinearity

*** p<0.01,

** p<0.05,

* p<0.1

**Table 4:** Household vitamin A-rich fruits and vegetables consumption and production.

	(1) Consume 6–11 mo.	(2) Consume 12–17 mo.	(3) Consume 18–23 mo.	(4) Consume 6–23 mo.	(5) Consume 24–59 mo.
HH produces FVA	0.711	0.608	1.238***	0.981***	0.445
	(1.19)	(0.75)	(0.48)	(0.36)	(0.30)
Wealth quintile	0.086	0.156	0.184*	0.150	0.198**
	(0.26)	(0.18)	(0.11)	(0.12)	(0.10)
Produce FVA X Wealth quintile	-0.202	-0.094	-0.270*	-0.209**	-0.146*
	(0.39)	(0.23)	(0.16)	(0.10)	(0.08)
Land rented/used (hectares)	-0.171	-0.065	0.026	-0.035	0.022
	(0.23)	(0.14)	(0.06)	(0.10)	(0.02)
Land owned only (hectares)	0.210	-0.157	0.008	-0.009	0.000
	(0.19)	(0.15)	(0.02)	(0.02)	(0.01)
Child (female)	0.236	0.086	-0.073	0.036	0.173*
	(0.18)	(0.25)	(0.19)	(0.11)	(0.10)
Whether breastfed	-0.797	0.319	-0.244	-0.514***	0.061
	(0.95)	(0.66)	(0.24)	(0.17)	(0.08)
Mother’s age (years)	0.064	0.042*	0.018	0.036***	0.001
	(0.05)	(0.02)	(0.02)	(0.01)	(0.01)
Mother’s schooling (years)	0.299	0.285	0.035	0.105	-0.065
	(0.23)	(0.23)	(0.12)	(0.11)	(0.04)
Mother’s schooling (sq.)	-0.018	-0.015	-0.003	-0.007	0.008***
	(0.01)	(0.02)	(0.01)	(0.01)	(0.00)
Mother can read	-0.861	-0.722	0.056	-0.167	0.033
	(0.75)	(0.91)	(0.42)	(0.34)	(0.21)
Mother’s BMI (Kg/m^2^)	-0.027	-0.004	0.036	0.001	-0.003
	(0.06)	(0.06)	(0.04)	(0.02)	(0.02)
Female head of household	-0.116	-0.409	-0.229	-0.241**	-0.287***
	(0.27)	(0.37)	(0.23)	(0.11)	(0.10)
Excluded caste	0.089	0.083	-0.239	-0.221	-0.257
	(0.42)	(0.46)	(0.27)	(0.28)	(0.17)
Non-Hindu	-0.006	0.807	-0.050	0.049	0.156
	(0.36)	(0.52)	(0.43)	(0.32)	(0.15)
Hill	11.838***	13.053***	13.728***	13.868***	3.103***
	(1.11)	(1.28)	(1.13)	(1.10)	(0.12)
Terai	13.957***	15.473***	17.486***	16.528***	6.216***
	(1.07)	(1.20)	(1.06)	(1.06)	(0.07)
Year (2014)	+	0.116	-0.564**	0.059	-0.237
		(0.99)	(0.22)	(0.23)	(0.20)
Constant	-14.966***	-17.200***	-17.719***	-16.983***	-5.124***
	(2.97)	(1.72)	(1.44)	(1.26)	(0.65)
Observations	389	381	800	1,635	4,343
VDC FE	Yes	Yes	Yes	Yes	Yes

Unit of observation is an individual child between 6–59 months. Coefficients are log-odds ratios. Standard errors in parentheses, clustered on VDCs. All results are from weighted logit regressions with fixed effects for each of 21 VDCs and 2 years. Survey weights are used for children in the balanced panel. The weights are 0.537 for Mountain, 1.711 for Hill and 0.834 for Terai. Description of variables: Fruits and vegetables rich in vitamin A (FVA) produced is a dummy = 1 if households produce FVA and 0 otherwise. Whether breastfed is dummy = 1 if children were breastfed in the past seven days and 0 otherwise. Excluded castes are dummy = 1 if Dalit, Janajati and other Terai caste and 0 otherwise. Non-Hindu are dummy = 1 if a household is non-Hindu and 0 if Hindu. Hill and Terai are dummy = 1 and 0 otherwise and dropped if dummy = 1 for Mountain. VDCs with no variation in outcome fall out of the regression.

^+^ Dropped because of collinearity

*** p<0.01,

** p<0.05,

* p<0.1

**Table 5:** Household fruit and vegetable production and consumption.

	(1) Consume 6–11 mo.	(2) Consume 12–17 mo.	(3) Consume 18–23 mo.	(4) Consume 6–23 mo.	(5) Consume 24–59 mo.
HH produces FV	1.157	0.504	0.730	0.563	1.084***
	(0.76)	(0.86)	(0.52)	(0.42)	(0.35)
Wealth quintile	0.150	0.226	0.104	0.071	0.098
	(0.21)	(0.24)	(0.18)	(0.13)	(0.14)
Produce FV X Wealth quintile	-0.261	-0.191	-0.187	-0.122	-0.237*
	(0.27)	(0.29)	(0.19)	(0.12)	(0.13)
Land rented/used (hectares)	-0.429	0.302	0.104**	0.036	0.056
	(0.43)	(0.36)	(0.05)	(0.05)	(0.04)
Land owned only (hectares)	-0.259	0.030	0.010	-0.023	0.028
	(0.18)	(0.11)	(0.02)	(0.02)	(0.02)
Child (female)	0.083	0.795***	-0.218	0.129	0.081
	(0.23)	(0.21)	(0.18)	(0.12)	(0.12)
Whether breastfed	-0.067	-1.018	-0.081	-0.953***	0.007
	(1.11)	(0.66)	(0.41)	(0.25)	(0.08)
Mother’s age (years)	0.037	0.065*	0.042**	0.049***	0.043***
	(0.02)	(0.04)	(0.02)	(0.01)	(0.01)
Mother’s schooling (years)	0.052	0.250	0.009	0.140*	0.001
	(0.15)	(0.34)	(0.14)	(0.08)	(0.08)
Mother’s schooling (sq.)	-0.002	-0.013	0.011	-0.008	0.006
	(0.01)	(0.02)	(0.01)	(0.01)	(0.01)
Mother can read	0.182	0.305	-0.526	-0.138	0.208
	(0.77)	(0.81)	(0.42)	(0.24)	(0.22)
Mother’s BMI (Kg/m^2^)	0.008	-0.131*	0.070	-0.027	0.054**
	(0.05)	(0.08)	(0.06)	(0.03)	(0.02)
Female head of household	0.221	-0.173	-0.137	0.026	0.019
	(0.34)	(0.33)	(0.33)	(0.15)	(0.14)
Excluded caste	0.193	0.975***	-0.310	0.089	0.056
	(0.36)	(0.37)	(0.32)	(0.17)	(0.17)
Non-Hindu	0.259	-0.520	-1.141**	-0.516	-0.249
	(0.52)	(0.44)	(0.55)	(0.38)	(0.25)
Hill	2.872***	-0.744	3.296***	2.208***	1.835***
	(0.40)	(0.64)	(0.67)	(0.44)	(0.27)
Terai	0.294	-0.098	1.947***	0.840***	2.228***
	(0.32)	(0.48)	(0.28)	(0.24)	(0.14)
Year (2014)	+	0.359	-0.489***	0.812***	-0.313
		(0.76)	(0.19)	(0.25)	(0.21)
Constant	-2.348	2.097	-2.029	-0.198	-2.252***
	(1.48)	(1.90)	(1.31)	(0.73)	(0.63)
Observations	376	381	796	1,609	4,321
VDC FE	Yes	Yes	Yes	Yes	Yes

Unit of observation is an individual child between 6–59 months. Coefficients are log-odds ratios. Standard errors in parentheses, clustered on VDCs. All results are from weighted logit regressions with fixed effects for each of 21 VDCs and 2 years. Survey weights are used for children in the balanced panel. The weights are 0.537 for Mountain, 1.711 for Hill and 0.834 for Terai. Description of variables: Fruits and vegetables (FVs) produced is a dummy = 1 if households produce FVs and 0 otherwise. Whether breastfed is dummy = 1 if children were breastfed in the past seven days and 0 otherwise. Excluded castes are dummy = 1 if Dalit, Janajati and other Terai caste and 0 otherwise. Non-Hindu are dummy = 1 if a household is non-Hindu and 0 if Hindu. Hill and Terai are dummy = 1 and 0 otherwise and dropped if dummy = 1 for Mountain. VDCs with no variation in outcome fall out of the regression.

^+^ Dropped because of collinearity

*** p<0.01,

** p<0.05,

* p<0.1

**Table 6:** Household meat production and consumption.

	(1) Consume 6–11 mo.	(2) Consume 12–17 mo.	(3) Consume 18–23 mo.	(4) Consume 6–23 mo.	(5) Consume 24–59 mo.
HH produces meat	1.826	-1.761*	0.866	0.123	0.208
	(1.20)	(0.90)	(0.54)	(0.29)	(0.17)
Wealth quintile	0.414	-0.119	0.198**	0.139*	0.187***
	(0.28)	(0.20)	(0.10)	(0.07)	(0.07)
Produce meat X Wealth quintile	-0.467	0.433**	-0.206	-0.059	-0.087**
	(0.30)	(0.22)	(0.14)	(0.09)	(0.04)
Land rented/used (hectares)	-1.366***	-0.314**	-0.044**	-0.091***	-0.027
	(0.45)	(0.16)	(0.02)	(0.03)	(0.02)
Land owned only (hectares)	-0.107	-0.049	0.030	0.023	0.013
	(0.25)	(0.09)	(0.03)	(0.03)	(0.01)
Child (female)	0.833***	-0.079	0.124	0.126	0.137**
	(0.28)	(0.23)	(0.15)	(0.13)	(0.06)
Whether breastfed	-3.271***	-0.457	-0.469	-0.817***	-0.110
	(1.23)	(0.87)	(0.35)	(0.23)	(0.08)
Mother’s age (years)	0.030	0.023	-0.007	0.010	-0.008
	(0.03)	(0.03)	(0.02)	(0.01)	(0.01)
Mother’s schooling (years)	0.180	0.084	0.150	0.124**	0.057
	(0.17)	(0.23)	(0.10)	(0.06)	(0.07)
Mother’s schooling (sq.)	-0.023**	-0.011	-0.010	-0.011***	-0.006
	(0.01)	(0.01)	(0.01)	(0.00)	(0.00)
Mother can read	0.292	0.066	-0.421	-0.239	0.116
	(0.52)	(0.89)	(0.32)	(0.25)	(0.28)
Mother’s BMI (Kg/m^2^)	0.013	0.100	0.075**	0.059*	0.032***
	(0.04)	(0.06)	(0.04)	(0.03)	(0.01)
Female head of household	-0.553	-0.004	-0.585***	-0.280*	-0.218***
	(0.53)	(0.31)	(0.19)	(0.16)	(0.07)
Excluded caste	-0.596*	0.226	-0.159	-0.208*	-0.123
	(0.35)	(0.34)	(0.22)	(0.13)	(0.18)
Non-Hindu	1.131	0.921*	0.626*	0.627**	0.398
	(0.80)	(0.49)	(0.34)	(0.24)	(0.25)
Hill	1.389***	13.905***	1.733***	1.629***	1.055***
	(0.48)	(1.06)	(0.31)	(0.17)	(0.14)
Terai	0.572*	13.446***	1.164***	0.942***	0.878***
	(0.33)	(1.08)	(0.23)	(0.12)	(0.09)
Year (2014)	+	0.075	-0.088	0.469***	-0.214**
		(0.64)	(0.13)	(0.16)	(0.11)
Constant	-0.438	-15.179***	-2.294***	-2.104***	-1.085***
	(1.68)	(2.49)	(0.59)	(0.67)	(0.19)
Observations	396	394	800	1,635	4,343
VDC FE	Yes	Yes	Yes	Yes	Yes

Unit of observation is an individual child between 6–59 months. Coefficients are log-odds ratios. Standard errors in parentheses, clustered on VDCs. All results are from weighted logit regressions with fixed effects for each of 21 VDCs and 2 years. Survey weights are used for children in the balanced panel. The weights are 0.537 for Mountain, 1.711 for Hill and 0.834 for Terai. Description of variables: Meat produced is a dummy = 1 if households own meat-producing livestock and 0 otherwise. Whether breastfed is dummy = 1 if children were breastfed in the past seven days and 0 otherwise. Excluded castes are dummy = 1 if Dalit, Janajati and other Terai caste and 0 otherwise. Non-Hindu are dummy = 1 if a household is non-Hindu and 0 if Hindu. Hill and Terai are dummy = 1 and 0 otherwise and dropped if dummy = 1 for Mountain. VDCs with no variation in outcome fall out of the regression.

^+^ Dropped because of collinearity

*** p<0.01,

** p<0.05,

* p<0.1

**Table 7:** Household egg production and consumption.

	(1) Consume 6–11 mo.	(2) Consume 12–17 mo.	(3) Consume 18–23 mo.	(4) Consume 6–23 mo.	(5) Consume 24–59 mo.
HH produces egg	0.778	0.223	1.636***	1.315***	0.978***
	(1.27)	(0.97)	(0.51)	(0.46)	(0.30)
Wealth quintile	0.684***	0.368**	0.150*	0.273***	0.206***
	(0.26)	(0.17)	(0.09)	(0.09)	(0.06)
Produce egg X Wealth quintile	-0.074	0.253	-0.131	-0.127	-0.096
	(0.28)	(0.21)	(0.18)	(0.14)	(0.08)
Land rented/used (hectares)	-0.619	-1.072***	0.008	-0.076	-0.015*
	(0.64)	(0.40)	(0.05)	(0.06)	(0.01)
Land owned only (hectares)	-2.428***	0.095	-0.011	-0.023	-0.005
	(0.64)	(0.09)	(0.01)	(0.02)	(0.01)
Child (female)	-0.185	0.395	-0.315*	-0.162	-0.019
	(0.34)	(0.38)	(0.17)	(0.14)	(0.11)
Whether breastfed	-2.030**	-0.379	-0.389**	-0.614***	-0.008
	(1.01)	(0.79)	(0.19)	(0.19)	(0.09)
Mother’s age (years)	0.053	0.032	0.015	0.034*	-0.011
	(0.04)	(0.04)	(0.02)	(0.02)	(0.01)
Mother’s schooling (years)	0.222	0.170	0.013	0.096	0.033
	(0.31)	(0.27)	(0.08)	(0.07)	(0.05)
Mother’s schooling (sq.)	-0.018	-0.005	-0.004	-0.007*	-0.001
	(0.02)	(0.01)	(0.01)	(0.00)	(0.00)
Mother can read	-0.628	-0.685	0.681**	-0.031	0.143
	(1.17)	(1.17)	(0.32)	(0.42)	(0.23)
Mother’s BMI (Kg/m^2^)	0.027	0.029	0.036	0.022	0.026**
	(0.06)	(0.05)	(0.03)	(0.02)	(0.01)
Female head of household	-0.103	-0.444	0.146	-0.018	-0.006
	(0.43)	(0.50)	(0.27)	(0.22)	(0.13)
Excluded caste	-0.257	-0.110	-0.548**	-0.380*	-0.418**
	(0.42)	(0.47)	(0.25)	(0.20)	(0.18)
Non-Hindu	0.294	0.885	0.525*	0.331	0.363**
	(0.72)	(0.69)	(0.27)	(0.21)	(0.18)
Hill	-1.033	13.275***	-0.055	0.265	1.189***
	(0.80)	(1.28)	(0.22)	(0.22)	(0.14)
Terai	-0.587	10.484***	-0.093	-0.432***	0.949***
	(0.44)	(1.16)	(0.11)	(0.13)	(0.08)
Year (2014)	+	0.858*	-0.321	0.314**	-0.239**
		(0.51)	(0.24)	(0.15)	(0.11)
Constant	-3.635**	-16.729***	-3.036***	-3.667***	-3.115***
	(1.61)	(1.90)	(0.88)	(0.74)	(0.33)
Observations	350	399	796	1,631	4,343
VDC FE	Yes	Yes	Yes	Yes	Yes

Unit of observation is an individual child between 6–59 months. Coefficients are log-odds ratios. Standard errors in parentheses, clustered on VDCs. All results are from weighted logit regressions with fixed effects for each of 21 VDCs and 2 years. Survey weights are used for children in the balanced panel. The weights are 0.537 for Mountain, 1.711 for Hill and 0.834 for Terai. Description of variables: Eggs produced is a dummy = 1 if households own eggs-producing livestock and 0 otherwise. Whether breastfed is dummy = 1 if children were breastfed in the past seven days and 0 otherwise. Excluded castes are dummy = 1 if Dalit, Janajati and other Terai caste and 0 otherwise. Non-Hindu are dummy = 1 if a household is non-Hindu and 0 if Hindu. Hill and Terai are dummy = 1 and 0 otherwise and dropped if dummy = 1 for Mountain. VDCs with no variation in outcome fall out of the regression.

^+^ Dropped because of collinearity

*** p<0.01,

** p<0.05,

* p<0.1

**Table 8:** Household dairy production and consumption.

	(1) Consume 6–11 mo.	(2) Consume 12–17 mo.	(3) Consume 18–23 mo.	(4) Consume 6–23 mo.	(5) Consume 24–59 mo.
HH produces dairy	-1.706***	-0.490	0.982***	0.055	1.203***
	(0.48)	(0.50)	(0.31)	(0.28)	(0.29)
Wealth quintile	-0.268*	-0.016	0.361***	0.114	0.416***
	(0.16)	(0.11)	(0.11)	(0.08)	(0.08)
Produce dairy X Wealth quintile	0.532***	0.209	-0.281**	0.013	-0.256***
	(0.17)	(0.15)	(0.11)	(0.10)	(0.09)
Land rented/used (hectares)	-0.611**	-0.325*	0.041	-0.025	-0.018
	(0.30)	(0.18)	(0.06)	(0.03)	(0.02)
Land owned only (hectares)	-0.109	0.292	0.036	0.012	0.006
	(0.08)	(0.25)	(0.04)	(0.02)	(0.01)
Child (female)	-0.120	-0.277	-0.050	-0.147	-0.059
	(0.22)	(0.22)	(0.17)	(0.12)	(0.10)
Whether breastfed	-0.193	-1.467*	-0.901***	-0.984***	-0.045
	(0.89)	(0.83)	(0.23)	(0.29)	(0.09)
Mother’s age (years)	0.042	0.015	0.000	0.012	0.001
	(0.04)	(0.02)	(0.02)	(0.01)	(0.01)
Mother’s schooling (years)	-0.013	-0.112	0.153	0.063	0.107***
	(0.15)	(0.19)	(0.11)	(0.05)	(0.03)
Mother’s schooling (sq.)	0.005	0.008	-0.009	-0.002	-0.002
	(0.01)	(0.01)	(0.01)	(0.00)	(0.00)
Mother can read	0.723	0.733	0.133	0.253	0.018
	(0.53)	(0.78)	(0.36)	(0.23)	(0.14)
Mother’s BMI (Kg/m^2^)	0.024	0.092**	-0.001	0.036**	0.044***
	(0.05)	(0.04)	(0.02)	(0.02)	(0.02)
Female head of household	-0.729**	0.168	-0.499**	-0.429**	-0.238***
	(0.31)	(0.45)	(0.23)	(0.18)	(0.06)
Excluded caste	-0.249	-0.278	-0.556	-0.446*	-0.593***
	(0.29)	(0.55)	(0.43)	(0.25)	(0.19)
Non-Hindu	-0.069	-0.431	-0.101	-0.303	-0.595***
	(0.43)	(0.58)	(0.44)	(0.37)	(0.22)
Hill	-2.100***	-1.390**	-2.944***	-2.096***	-1.290***
	(0.38)	(0.59)	(0.37)	(0.22)	(0.14)
Terai	-0.748**	0.969**	-0.773***	-0.324*	-0.242**
	(0.30)	(0.42)	(0.24)	(0.17)	(0.10)
Year (2014)	+	0.287	-0.212	0.041	-0.116
		(0.89)	(0.21)	(0.11)	(0.12)
Constant	0.817	-0.106	1.479**	0.915	-0.876*
	(0.93)	(0.76)	(0.63)	(0.60)	(0.48)
Observations	391	399	800	1,635	4,343
VDC FE	Yes	Yes	Yes	Yes	Yes

Unit of observation is an individual child between 6–59 months. Coefficients are log-odds ratios. Standard errors in parentheses, clustered on VDCs. All results are from weighted logit regressions with fixed effects for each of 21 VDCs and 2 years. Survey weights are used for children in the balanced panel. The weights are 0.537 for Mountain, 1.711 for Hill and 0.834 for Terai. Description of variables: Dairy produced is a dummy = 1 if households own dairy-producing livestock and 0 otherwise. Whether breastfed is dummy = 1 if children were breastfed in the past seven days and 0 otherwise. Excluded castes are dummy = 1 if Dalit, Janajati and other Terai caste and 0 otherwise. Non-Hindu are dummy = 1 if a household is non-Hindu and 0 if Hindu. Hill and Terai are dummy = 1 and 0 otherwise and dropped if dummy = 1 for Mountain. VDCs with no variation in outcome fall out of the regression.

^+^ Dropped because of collinearity

*** p<0.01,

** p<0.05,

* p<0.1

**Table 9:** Household legumes production and consumption.

	(1) Consume 6–11 mo.	(2) Consume 12–17 mo.	(3) Consume 18–23 mo.	(4) Consume 6–23 mo.	(5) Consume 24–59 mo.
HH produces legumes	0.084	0.469	-0.358	0.026	0.625
	(0.66)	(1.70)	(0.78)	(0.63)	(0.42)
Wealth quintile	0.087	0.027	0.383**	0.160*	0.540***
	(0.12)	(0.27)	(0.17)	(0.08)	(0.13)
Produces legumes X Wealth quintile	0.090	0.291	-0.014	0.059	-0.251
	(0.29)	(0.79)	(0.24)	(0.20)	(0.18)
Land rented/used (hectares)	0.208	-0.048	0.017	-0.002	-0.050
	(0.38)	(0.15)	(0.07)	(0.05)	(0.03)
Land owned only (hectares)	0.035	0.757	0.088	0.063	0.029
	(0.36)	(0.89)	(0.08)	(0.06)	(0.03)
Child (female)	-0.011	0.132	-0.171	0.018	-0.170
	(0.25)	(0.56)	(0.35)	(0.17)	(0.15)
Whether breastfed	0.171	+	-1.007***	-1.197***	0.134
	(1.55)		(0.37)	(0.41)	(0.18)
Mother’s age (years)	0.043	-0.005	0.035	0.035	0.014
	(0.05)	(0.05)	(0.05)	(0.03)	(0.01)
Mother’s schooling (years)	0.103	0.806	0.001	0.088	-0.159
	(0.26)	(0.55)	(0.18)	(0.15)	(0.18)
Mother’s schooling (sq.)	-0.009	-0.041	0.010	-0.002	0.016
	(0.02)	(0.03)	(0.01)	(0.01)	(0.01)
Mother can read	-0.395	-2.761*	-0.969	-0.615	0.733*
	(0.87)	(1.63)	(0.89)	(0.62)	(0.44)
Mother’s BMI (Kg/m^2^)	-0.060	0.339**	0.045	0.031	0.014
	(0.06)	(0.17)	(0.09)	(0.04)	(0.03)
Female head of household	0.699	0.525	-0.206	0.241	-0.566***
	(0.57)	(0.59)	(0.46)	(0.27)	(0.21)
Excluded caste	-0.271	0.015	-0.034	-0.160	-0.450
	(0.33)	(0.84)	(0.38)	(0.29)	(0.33)
Non-Hindu	-0.757	-0.551	0.588	-0.200	-0.450
	(0.53)	(0.99)	(0.96)	(0.52)	(0.36)
Hill	1.410***	-15.372***	-2.216***	-0.858*	-1.157***
	(0.51)	(1.11)	(0.66)	(0.44)	(0.34)
Terai	1.704***	-14.108***	-0.214	0.094	-0.166
	(0.42)	(1.19)	(0.47)	(0.20)	(0.20)
Year (2014)	+	1.636	-0.119	0.376	0.047
		(1.20)	(0.45)	(0.41)	(0.21)
Constant	0.086	8.129**	1.519	1.088	1.539**
	(1.78)	(3.66)	(2.25)	(1.66)	(0.76)
Observations	372	286	776	1,608	4,343
VDC FE	Yes	Yes	Yes	Yes	Yes

Unit of observation is an individual child between 6–59 months. Coefficients are log-odds ratios. Standard errors in parentheses, clustered on VDCs. All results are from weighted logit regressions with fixed effects for each of 21 VDCs and 2 years. Survey weights are used for children in the balanced panel. The weights are 0.537 for Mountain, 1.711 for Hill and 0.834 for Terai. Description of variables: Legumes produced is a dummy = 1 if households produce and 0 otherwise. Whether breastfed is dummy = 1 if children were breastfed in the past seven days and 0 otherwise. Excluded castes are dummy = 1 if Dalit, Janajati and other Terai caste and 0 otherwise. Non-Hindu are dummy = 1 if a household is non-Hindu and 0 if Hindu. Hill and Terai are dummy = 1 and 0 otherwise and dropped if dummy = 1 for Mountain. VDCs with no variation in outcome fall out of the regression.

^+^ Dropped because of collinearity

*** p<0.01,

** p<0.05,

* p<0.1

**Table 10:** Diet diversity and number of food groups produced by child's age (unbalanced panel).

	(1) MDDC ≥4 6–11 mo.	(2) MDDC ≥4 12–17 mo.	(3) MDDC ≥4 18–23 mo.	(4) MDDC ≥4 6–23 mo.	(5) MDDC ≥4 24–59 mo.
Food group grown (0–7)	0.083	-0.131	0.335***	0.052	0.166**
	(0.09)	(0.13)	(0.10)	(0.07)	(0.08)
Wealth quintile (1–5)	0.211*	-0.070	0.559***	0.160	0.369**
	(0.12)	(0.21)	(0.21)	(0.11)	(0.18)
Wealth quintile X Food group grown	-0.031	0.093**	-0.096***	-0.007	-0.011
	(0.03)	(0.04)	(0.03)	(0.02)	(0.03)
Land rented/used (hectares)	0.022	-0.081*	-0.028	-0.018	-0.017**
	(0.02)	(0.05)	(0.03)	(0.01)	(0.01)
Land owned only (hectares)	0.000	0.067***	0.080**	0.022	0.025
	(0.01)	(0.02)	(0.04)	(0.02)	(0.02)
Child (female)	0.060	0.210	0.004	0.051	-0.006
	(0.17)	(0.23)	(0.17)	(0.11)	(0.07)
Whether breastfed	0.080	-1.647**	-0.810**	-1.485***	-0.050
	(0.59)	(0.68)	(0.32)	(0.11)	(0.06)
Mother’s age (years)	0.007	0.028	0.029	0.027***	0.011
	(0.02)	(0.02)	(0.02)	(0.01)	(0.01)
Mother’s schooling (years)	0.046	0.046	-0.025	0.041	-0.036
	(0.07)	(0.10)	(0.16)	(0.04)	(0.06)
Mother’s schooling (squared)	0.001	-0.000	0.008	0.000	0.007
	(0.01)	(0.01)	(0.01)	(0.00)	(0.00)
Mother can read	0.043	-0.074	0.259	0.040	0.372
	(0.34)	(0.59)	(0.48)	(0.22)	(0.25)
Mother’s BMI (Kg/m^2^)	-0.053	0.089**	-0.017	-0.023	0.035**
	(0.04)	(0.04)	(0.05)	(0.02)	(0.02)
Female head of household	-0.130	-0.265	-0.158	-0.142*	-0.307***
	(0.19)	(0.21)	(0.29)	(0.08)	(0.06)
Excluded caste	-0.567***	-0.134	-0.679***	-0.463***	-0.478***
	(0.14)	(0.19)	(0.19)	(0.11)	(0.16)
Non-Hindu	0.009	0.217	0.068	-0.036	0.083
	(0.26)	(0.29)	(0.27)	(0.19)	(0.22)
Hill	1.228***	-0.204	0.085	0.493***	0.559***
	(0.19)	(0.37)	(0.29)	(0.16)	(0.11)
Terai	1.054***	1.941***	1.681***	1.211***	2.319***
	(0.19)	(0.26)	(0.25)	(0.12)	(0.13)
Year (2014)	-0.211*	-0.334*	-0.533***	-0.260***	-0.351***
	(0.11)	(0.19)	(0.17)	(0.10)	(0.10)
Constant	-0.286	-1.432	-0.532	0.809	-1.550**
	(0.75)	(1.77)	(0.90)	(0.61)	(0.66)
Observations	1,034	934	1,040	3,033	6,213
VDC FE	Yes	Yes	Yes	Yes	Yes

Unit of observation is an individual child between 6–59 months. Coefficients are log-odds ratios. Standard errors in parentheses, clustered on VDCs. All results are from weighted logit regressions with fixed effects for each of 21 VDCs and 2 years. Survey weights are used for children in the unbalanced panel. The weights are 0.537 for Mountain, 1.711 for Hill and 0.834 for Terai. Description of variables: Food groups (1–7) grown correspond to seven food group based on WHO and UNICEF Minimum Dietary Diversity for Children. Non-farming households (food group = 0) are also included. Whether breastfed is dummy = 1 if children were breastfed in the past seven days and 0 otherwise. Excluded castes are dummy = 1 if Dalit, Janajati and other Terai caste and 0 otherwise. Non-Hindu are dummy = 1 if a household is non-Hindu and 0 if Hindu. Hill and Terai are dummy = 1 and 0 otherwise and dropped if dummy = 1 for Mountain.

*** p<0.01,

** p<0.05,

* p<0.1

**Table 11:** Diet diversity and agricultural diversity quintile produced by child's age (unbalanced panel).

	(1) MDDC ≥4 6–11 mo.	(2) MDDC ≥4 12–17 mo.	(3) MDDC ≥4 18–23 mo.	(4) MDDC ≥4 6–23 mo.	(5) MDDC ≥4 24–59 mo.
Ag. diversity quintile (0–5)	0.223**	0.002	0.496***	0.153*	0.306***
	(0.11)	(0.20)	(0.17)	(0.09)	(0.10)
Wealth quintile (1–5)	0.248**	0.084	0.548***	0.205*	0.380**
	(0.12)	(0.22)	(0.21)	(0.12)	(0.17)
Ag. diversity quintile X Wealth quintile	-0.057	0.077	-0.142***	-0.028	-0.019
	(0.04)	(0.07)	(0.05)	(0.03)	(0.04)
Land rented/used (hectares)	0.020	-0.085*	-0.030	-0.019	-0.023***
	(0.02)	(0.05)	(0.03)	(0.01)	(0.01)
Land owned only (hectares)	0.000	0.070***	0.079**	0.022	0.020
	(0.01)	(0.02)	(0.03)	(0.02)	(0.02)
Child (female)	0.057	0.212	0.013	0.054	-0.006
	(0.17)	(0.22)	(0.17)	(0.11)	(0.07)
Whether breastfed	0.039	-1.598**	-0.818**	-1.483***	-0.050
	(0.60)	(0.64)	(0.32)	(0.11)	(0.06)
Mother’s age (years)	0.006	0.025	0.030	0.026***	0.010
	(0.02)	(0.02)	(0.02)	(0.01)	(0.01)
Mother’s schooling (years)	0.049	0.040	-0.034	0.038	-0.044
	(0.07)	(0.10)	(0.16)	(0.04)	(0.06)
Mother’s schooling (sq.)	0.000	0.000	0.009	0.000	0.007*
	(0.01)	(0.01)	(0.01)	(0.00)	(0.00)
Mother can read	-0.003	-0.058	0.263	0.029	0.364
	(0.34)	(0.60)	(0.49)	(0.22)	(0.26)
Mother’s BMI (Kg/m^2^)	-0.053	0.091**	-0.018	-0.023	0.038**
	(0.04)	(0.04)	(0.05)	(0.02)	(0.02)
Female head of household	-0.090	-0.233	-0.118	-0.115	-0.275***
	(0.20)	(0.20)	(0.32)	(0.08)	(0.06)
Excluded caste	-0.554***	-0.059	-0.656***	-0.449***	-0.401***
	(0.13)	(0.24)	(0.17)	(0.10)	(0.14)
Non-Hindu	0.020	0.219	0.098	-0.031	0.130
	(0.26)	(0.30)	(0.27)	(0.18)	(0.21)
Hill	1.289***	-0.156	0.132	0.543***	0.631***
	(0.20)	(0.39)	(0.30)	(0.16)	(0.12)
Terai	1.198***	2.115***	1.755***	1.301***	2.524***
	(0.20)	(0.23)	(0.30)	(0.11)	(0.13)
Year (2014)	-0.221**	-0.338*	-0.498***	-0.258***	-0.346***
	(0.11)	(0.18)	(0.17)	(0.10)	(0.10)
Constant	-0.610	-2.087	-0.586	0.555	-1.909***
	(0.66)	(1.74)	(0.91)	(0.58)	(0.58)
Observations	1,034	934	1,040	3,033	6,213
VDC FE	Yes	Yes	Yes	Yes	Yes

Unit of observation is an individual child between 6–59 months. Coefficients are log-odds ratios. Standard errors in parentheses, clustered on VDCs. All results are from weighted logit regressions with fixed effects for each of 21 VDCs and 2 years. Survey weights are used for children in the unbalanced panel. The weights are 0.537 for Mountain, 1.711 for Hill and 0.834 for Terai. Description of variables: Agricultural production diversity (Ag. div) quintile (1–5) is generated from total count of food species (1–32) produced in a farming household; non-farmers (food quintile = 0) are also included. Food species count is created from a sum of crop species (0–29) and livestock grown (0–6) per household. Range of food species count included in the quintile is as follows: First (1–2), Second (3–5), Third (6–9), Fourth (10–14), and Fifth (15–32). Whether breastfed is dummy = 1 if children were breastfed in the past seven days and 0 otherwise. Excluded castes are dummy = 1 if Dalit, Janajati and other Terai caste and 0 otherwise. on-Hindu are dummy = 1 if a household is non-Hindu and 0 if Hindu. Hill and Terai are dummy = 1 and 0 otherwise and dropped if dummy = 1 for Mountain.

*** p<0.01,

** p<0.05,

* p<0.1

The mediating effect of child age on the association between production of individual food groups and its consumption is quantified using logit regression models. Almost all children consume starchy staples, so results are reported for the remaining six nutrient-dense food groups using the following base specification:

$${Cnsmptn}_{i}\;=\;\beta_{0}+\beta_{1}{prdctn}_{ih}+\beta_{2}{wealth}_{ih}+\beta_{3}{prdctn}_{ih}\times {wealth}_{ih}+\delta Z_{i}+\alpha {VDC}_{i}+\gamma year+\mu_{i\;}$$ (4)

The base specification is run for each of the six WHO-determined food groups, where Cnsmptn_*i*_ and prdctn_*ih*_ indicate binary variables. Cnsmptn_*i*_ indicates whether child *i* eats a food group and prdctn_*ih*_ represents whether household *h* of that child grows the same food group. Note that production and consumption may refer to different foods within that group, to allow for the possibility of exchanging one food for another within that group (for example, trading carrots for tomatoes) as well as self-provisioning of each food. As noted above, *β*_1_ is the primary coefficient of interest. *β*_1_ tests the null hypothesis that there is no difference in child’s intake of that food group between households that do and do not produce it. *β*_3_ shows the coefficient of the interaction between a food group produced and wealth quintile of household *h* of a child *i*. Coefficients are intended for hypothesis testing rather than estimation, but when significantly different from zero the magnitude of these log-odds ratios, when exponentiated, provide an estimate of the probability that a child’s intake includes that food group or meets the MDDS minimum. The remainder of the notations, functional forms and control variables are the same as those described in Eqs 1–3.

## Results

### Agricultural production diversity and minimum dietary diversity score for children (MDDC)

Our principal finding is a significant association (p<0.001) between children reaching the minimum dietary diversity score (≥4 in the previous week) and the number of food groups grown in the household for all children over 24 months, and for children in the poorest households from 18–23 months of age, but not for children aged 6–23 months or in the 6–11 or 12–18 month groups (Table 1).

All else equal, for each additional food group that households produce, log-odds of meeting the minimum dietary diversity score (≥4) increases 0.253 (p = 0.01) among children between the ages of 24–59 months. In the smaller sample of younger children aged 18–23 months, there is also a significant effect but only for the poorest households: in the lowest quintile of wealth the effect is 0.43 minus 0.137 (both p = 0.01). At higher levels of household wealth and hence purchasing power, for children 18–23 months the association between achieving minimum dietary diversity and number of food groups grown on the family farm goes to zero—and it is zero at all levels of wealth for children under 18 of age, whose diets require more labor-intensive specialized feeding practices [9]. Each of the narrow age subgroups have relatively small sample sizes: n = 396 for 6–11 month-olds, and n = 399 for 12–17 month-olds. This is why we perform the same test for the larger 6–23 month group (n = 1,635) and also for the unbalanced sample that includes children observed in only one of the two years (n = 1,034 for 6–11 month-olds, and n = 934 for 12–17 month-olds) (Table 10).

Our alternative measure of production diversity counts each crop or livestock species separately to consider agroecological diversity even within food groups, and then transform the number of species grown into quintiles of the distribution for comparability with number of food groups or quintiles of wealth. Results are similar to our main findings, with agricultural diversity significantly associated with achieving minimum dietary diversity only after two years of age, and below that only in the poorer households (Table 2). For every unit increase in agricultural diversity quintile, log-odds of meeting minimum dietary diversity score (≥4) increases by 0.361 (p = 0.01) for children aged 24–59 months, while for children aged 18–23 months or 6–23 months the effect is significant only in the poorer households. The coefficient estimate for 6–11 months is statistically significant as well, but that sample is relatively small (n = 396). Further to our main results in Tables 1 and 2, we find that farming as such is not significantly associated with meeting minimum dietary diversity in any age category (Table 3). We also compare these results to the larger unbalanced panel, with attrition and replacement between rounds of the survey, using the household’s number of food groups produced (Table 10) and their quintile of agricultural diversity (Table 11).

Among control variables in addition to household wealth, the most consistently significant factors associated with achieving minimum dietary diversity are whether the child is still breastfeeding (-), the mother’s educational attainment (+), being from an excluded caste (-), and living in the Terai region (+), with additional significance in some regressions of the mother’s age (+), BMI (+) and having a female head of household (-). These findings are broadly similar to previous studies [36,37,38,39].

The negative association between breastfeeding and solid food intake arises primarily in older infancy, e.g. for the 18–23 month olds. In that age range, breastmilk and solid foods are likely to substitute for each other as sources of energy and nutrients [39,40,41], with diversity in IYCF practices due to differences in attitude, behaviors and perceptions on infant feeding [42,43].The positive association we find with maternal schooling is typical of previous work [9,44], as is the negative association with being from a socially excluded caste such as Dalit, Janajati, and Terai groups [31,45], except for those located in the Terai region which, controlling for the other factors mentioned above, is positively associated with meeting the minimum level of child dietary diversity. Without those controls, there is usually higher dietary diversity among children in Hill followed by Mountain and Terai [9,36].

### Individual food group production and consumption

Our tests for associations between children’s intake of each food group and their production on their family’s farm are presented in Tables 4–9. Of the six food groups considered to be nutrient dense, other than starchy staples, we find no such associations for legumes (Table 9) or meat (Table 6), implying that these can more readily be purchased if consumed (which occurs frequently for legumes, and rarely for meat). The strongest association is for eggs (Table 7), whose log odds of consumption is 0.98–1.6 points higher (p<0.01) when produced at home, for children both above and below two years of age, with no significance in the small samples of 6–11 and 12–17 months of age, and no significant interaction with household wealth.

For fruits and vegetables considered to be vitamin-A-rich (i.e., sources of provitamin A carotenoids), other fruits and vegetables, and dairy, there are significantly higher odds of intake in households that produce these foods on the family farm, but only at lower levels of wealth (Tables 4, 5 and 8). These could be the incremental food groups that contribute to those households reaching the minimum dietary diversity level reported in previous tables. These results hold primarily for older children. For those between the ages of 6–11 months and 12–17 months, the coefficients on fruits and vegetables (Tables 4 and 5) and eggs (Table 7) are positive, but not statistically significant. For the small sample of children aged 6–11 months we find a negative association between intake and dairy production on the farm (Table 8), which could be an anomaly due to the small sample involved (n = 391).

## Discussion and conclusions

Our principal finding is a strong association between the diversity of a household food production and dietary diversity among older children (18 or 24 months of age) in poorer households (the lowest one or two quintiles of wealth). Each additional food group produced is associated with 0.25 (p = 0.01) higher log-odds of meeting minimum dietary diversity score (≥4) by children aged 24–59 months. For younger children aged 18–23 months there is a similar effect but only in the poorest two quintiles of household wealth, and for infants 6–18 months we find little or no correlation between dietary intake and household production. Among the older children, the specific food groups for which intake is associated with production are eggs (at all wealth levels), and vitamin-A-rich fruits and vegetables, other fruits and vegetables, and dairy (in poorer households).

These findings control for geographic and climatic factors using village and year fixed effects, and also control for other variables that are significantly associated with intake notably the child’s breastfeeding status, the mother’s age, caste, educational attainment and BMI, and having a female head of household. By identifying differences among food groups and disaggregating by age we aim to help inform future interventions that could fill age-specific inadequacies in major food group dietary diversity across the preschool years in rural Nepal, to meet WHO recommendations regarding complementary feeding with foods such as dairy and eggs, as well as with nutrient-rich fruits and vegetables.

These findings suggest that interventions aimed at diversifying households’ farm production are likely to improve children’s diet quality only after they reach 18 months of age. To address poor nutrition among younger children may therefore require other actions such as targeted nutrition-specific interventions as well as improved market access to purchased complementary foods [46]. Even for older children, investments made in agricultural diversification are likely to be most effective if they target the poorest households least connected to food markets. We find that the upper three or four quintiles of Nepal’s income distribution, on average, have sufficient purchasing power for their market transactions to replace differences in what they grow. The exception in these data is eggs, for which the association between intake and production is not mediated by wealth.

Importantly, agricultural production data used here refer to crop and livestock raised at any time of year, while the dietary data refer to the previous seven days before the survey interview. As a result, many of the foods produced may be available only in particular seasons. This temporal limitation of household food production can sometimes be overcome by interventions that help households produce each food throughout the year, but local climate and resource constraints often make that prohibitively difficult. Since the timing of harvests usually differs over space, a more feasible approach to making intake more uniform over time is to facilitate market access. The effectiveness of food markets in overcoming local climate variation over time in Nepal is shown in Mulmi et al. (2016) [47]; similar effects of market access in smoothing nutritional outcomes has been shown for Africa in Darrouzet-Nardi and Masters (2017) [48].

A limitation of the study is the relatively small sample sizes for narrow age ranges of interest such as 6–11 months and 12–18 months. A potential extension of this work could focus on larger surveys with more children at each age. Another limitation is that our work addresses only the qualitative question of whether or not foods from a given group were consumed and produced. Future research should address the more complex question of how much of each food is consumed or produced. Another possible extension related to survey design is the potential to repeat surveys during the year to capture seasonal fluctuations, and to continue surveys for enough years for household and child fixed effects to be feasible, both of which would more fully exploit the panel nature of the survey.

Despite these limitations, the study provides clear insights regarding the heterogeneity in effects of home production on children’s intake of nutrient-dense foods, contributing to the rapidly growing evidence base on how and for whom agricultural interventions can best be used to improve child nutrition outcomes.
